# Case Report: Recurrent Clinical Symptoms of COVID-19 in Healthcare Professionals: A Series of Cases from Brazil

**DOI:** 10.4269/ajtmh.20-0893

**Published:** 2020-09-04

**Authors:** Christianne Fernandes Valente Takeda, Magda Moura de Almeida, Ricristhi Gonçalves de Aguiar Gomes, Tatiana Cisne Souza, Matheus Alves de Lima Mota, Luciano Pamplona de Góes Cavalcanti, Jeová Keny Baima Colares

**Affiliations:** 1 Hospital São José de Doenças Infecciosas (HSJ), Fortaleza, Brazil;; 2 Secretaria de Saúde do Estado do Ceará, Fortaleza, Brazil;; 3 Faculdade de Medicina da Universidade Federal do Ceará (FAMED/UFC), Fortaleza, Brazil;; 4 Universidade de Fortaleza (UNIFOR), Fortaleza, Brazil;; 5 Faculdade de Medicina do Centro Universitário Christus (UNICHRISTUS), Fortaleza, Brazil;; 6 Escola de Saúde Pública do Estado do Ceará, Fortaleza, Brazil

## Abstract

We describe six cases of healthcare professionals in Brazil who recovered but again presented symptoms consistent with COVID-19, with new positive reverse transcription (RT)-PCR test results. The cases reported herein presented symptom onset between March 16, 2020 and April 9, 2020. All were health professionals (four medical doctors), five were female, with a median age of 43.5 years, and three had comorbidities. All patients were confirmed for SARS-CoV-2 detection by RT-PCR in naso and/or oropharyngeal swab samples. Among the reported cases, three (50%) underwent RT-PCR testing in the period between the two symptomatic episodes, with negative results. The time elapsed between the onset of symptoms in the two episodes ranged from 53 to 70 days (median, 56.5 days). In the first episode, the main symptoms described were fever (4/6), myalgia (3/6), sore throat (3/6), and cough (3/6). Meanwhile, during the second episode, fever (4/6) and weakness (3/6) predominated. Most of the cases progressed without complications, although one individual presented hypoxemia (minimum SatO_2_ of 90%) in both episodes, and two, only in the second, one of which required intensive care unit admission, progressing with improvement after medication and receiving noninvasive ventilatory support. We report cases with recurrence of symptoms compatible with COVID-19, with positive RT-PCR results, that could represent the occurrence of viral reactivation or reinfection. The true nature of this phenomenon should be better clarified in future studies.

## INTRODUCTION

The new coronavirus SARS-CoV-2, the etiological agent of COVID-19, first emerged in Wuhan, China, in December 2019 and quickly spread throughout the world,^1,2^ with Brazil being one of the countries with the highest number of cases.^3^

Most cases evolve in a benign and self-limited manner. There is little definitive evidence for the development of immune protection after natural infection. In February 2020, four confirmed patients were reported, who were discharged after two negative reverse transcription (RT)-PCR tests, but reverted to being positive by RT-PCR 5–13 days after hospital discharge.^4^ Soon after that, several groups reported a similar phenomenon, which has received variable designations in the literature (recurrence, possible human reactivation, or reinfection by SARS-CoV-2).^1,5–7^

A distinct situation seems to happen when COVID-19 relapses with clinical manifestations of the disease associated with viral RNA detection on molecular testing, characterizing a condition compatible with the recurrence of COVID-19. A retrospective cohort of 55 individuals identified five cases (9%) that required readmission because of symptom recurrence between 4 and 17 days after discharge. Clinical progression was benign, with no differences observed from the acute phase.^8^ More recently, a case series was described involving 11 individuals in a similar situation, with reappearance of symptoms between 24 and 49 days after the beginning of the first episode. In this series, three deaths were reported, and viral detection was possible in cultured samples from one of the two recurrent cases tested.^9^ However, so far, there is no consensus on the possibility of recurrence, much less the distinction between possible human reactivation and reinfection by SARS-CoV-2.^5–7^

In Ceará, northeastern Brazil, more than 140,000 cases of COVID-19 have been reported, with more than 7,000 deaths,^10,11^ which has led the health system to collapse. Here, we describe six cases of healthcare professionals who recovered but again presented symptoms consistent with COVID-19, with new positive RT-PCR test results.

## CASE REPORTS

The cases reported herein presented symptom onset between March 16, 2020 and April 9, 2020. All were health professionals (four medical doctors), five were female, with a median age of 43.5 years, and three had comorbidities (Table 1). The cases presented were officially notified by the state epidemiological surveillance service of Ceará government. After information gathering, all patients agreed with the use of their anonymous medical data.

**Table 1:** Demographic aspects, signs, and symptoms of the cases of COVID-19 in Ceará, Brazil

Case	1	2	3	4	5	6
Age (gender)	29 (male)	63 (male)	40 (female)	67 (male)	47 (male)	31 (male)
Comorbidity	–	SAH	Ankylosing spondylitis and asthma	Obesity, SAH, obstructive sleep apnea syndrome, and rhinitis	–	–
DOS of first infection	March 16	March 16	March 18	March 20	March 23	April 9
Date of first RT-PCR	March 27	March 27	March 18	March 24	March 23	April 15
DOS of second infection	May 8	May 13	May 27	May 13	May 18	June 5
Date of second RT-PCR	May 13	May 18	June 1	May 16	May 22	June 8
Time between episodes	53	58	70	54	56	57
Time test: symptom 1	11	11	0	4	0	6
Time test: symptom 2	5	5	5	3	4	3
Adynamia	Yes	Yes	–	–	–	–
Chills	Yes	Yes	–	–	–	–
Myalgia	Yes	–	Yes	–	Yes	Yes
Arthralgia	–	–	Yes	Yes	–	–
Sore throat	Yes	Yes	–	–	–	–
Cough	Yes	–	Yes	–	Yes	–
Coryza	Yes	–	–	Yes	–	–
Headache	Yes	–	–	–	–	Yes
Asthenia	–	–	–	–	–	–
Anorexia	–	–	–	Yes	–	–
Diarrhea	–	–	–	Yes	–	–
Dyspnea	–	Yes	–	–	–	Yes
Fever	Yes	Yes	Yes	–	Yes	Yes
Oxygen saturation < 95%	–	–	–	–	–	Yes
Loss of sense of taste	Yes	–	–	–	–	–
Loss of sense of smell	Yes	–	Yes	–	–	–
Complications in the first episode	No	No	No	No	No	Hypoxemia (SatO_2_ 92%)
Complications in the second episode	No	Hypoxemia	No	Hypoxemia (intensive care unit)	No	Hypoxemia (SatO_2_ 90%)

DOS = date of onset of symptoms; RT-PCR = reverse transcription-PCR; SAH = systemic arterial hypertension.

**Figure 1.**
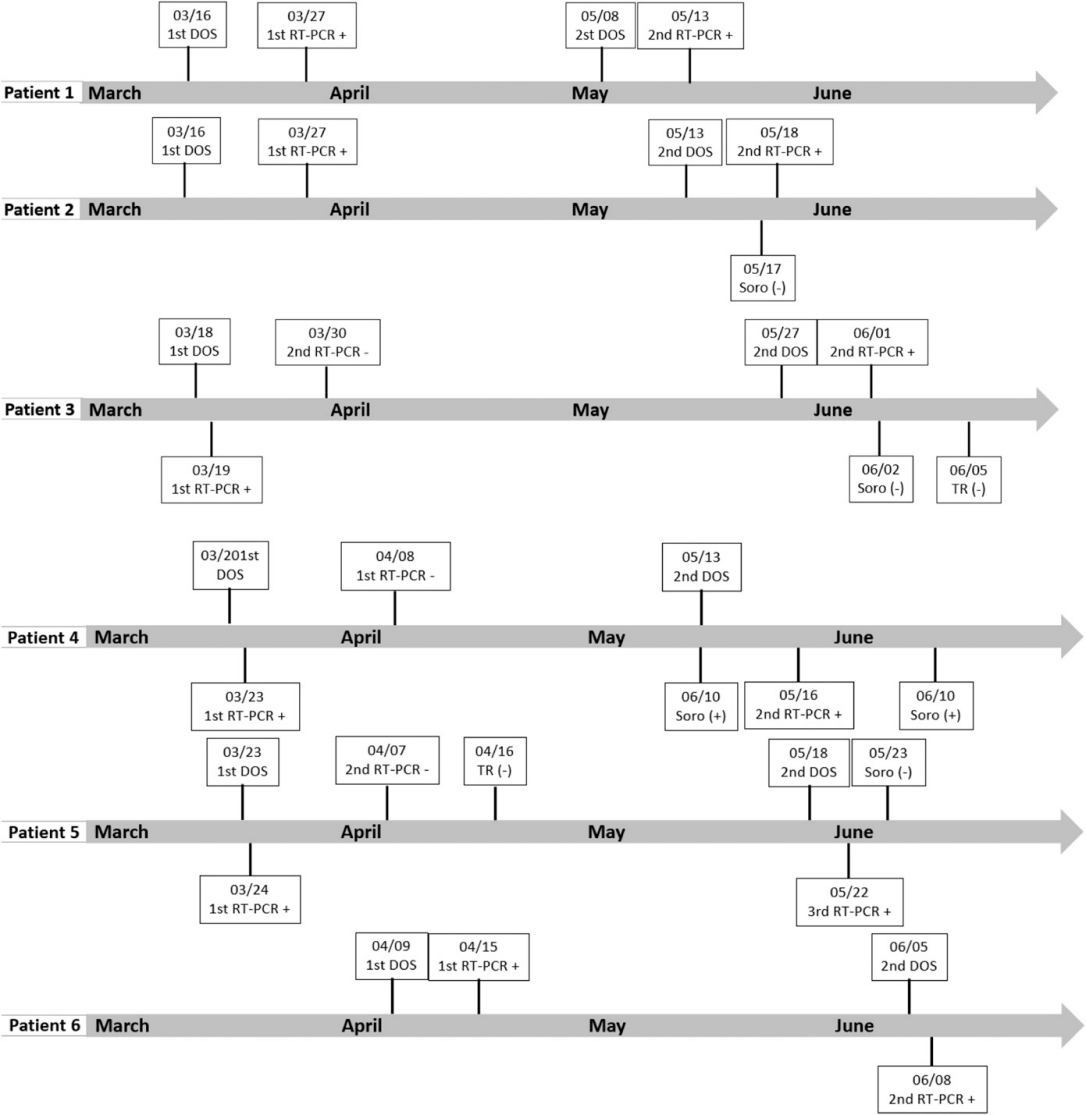
Time line of the confirmed cases in Ceará, Brazil. DOS = date of onset of symptoms; RT-PCR = Reverse transcription-PCR; + = positive; − = negative.

All patients were confirmed for SARS-CoV-2 detection by RT-PCR in naso and/or oropharyngeal swab samples, which were collected between 0 and 11 days after the first symptoms. Among the reported cases, 50% (3) underwent RT-PCR testing in the period between the two symptomatic episodes, with negative results. Only one of the patients underwent serological rapid testing in the same interval, which was also negative in two separate samples. In four cases, serological testing was also performed, but only after the second clinical episode, with negative initial results. Three cases later seroconverted positive by chemiluminescence microparticle immunoassay serology, collected on the 11th, the 28th, and the 37th day after the onset of symptoms in the second episode (Figure 1).

The time elapsed between the onset of symptoms in both episodes ranged from 53 to 70 days (median, 56.5 days). In the first episode, the main symptoms described were fever (4/6), myalgia (3/6), sore throat (3/6), and cough (3/6). Meanwhile, during the second episode, fever (4/6) and weakness (3/6) predominated. Myalgia and diarrhea were reported only in the first episode, but the highest number of symptoms was described during the second episode. In two cases, anosmia was observed in the second episode. Most of the cases progressed without complications, although one individual presented hypoxemia (minimum SatO_2_ of 90%) in both episodes, and two, only in the second, one of which required intensive care unit (ICU) admission, progressing with improvement after medication and receiving noninvasive ventilatory support.

During the first episode, patient 1 received oseltamivir for 10 days, and in the second, symptomatic medications, azithromycin and hydroxychloroquine, were prescribed, in addition to oseltamivir. Patient 2 was treated with symptomatic medication and oseltamivir in the first episode, whereas in the second, only symptomatic medication was prescribed. Patient 3 presented a high-resolution chest tomography (HRCT) with slight scattered and bilateral ground-glass pulmonary opacities, more evident in peripheral regions (less than 50% extension) and was treated with prednisone and azithromycin only in the second episode. Patient 4 required hospitalization only in the second episode, when he used hydroxychloroquine (800 mg on the first day, and then 400 mg/day for another 6 days), azithromycin (500 mg/day) for 5 days, and levofloxacin (750 mg/day) for 7 days. He evolved without improvement, being transferred to the ICU to receive high-flow oxygen therapy. He had HRCT with pulmonary involvement of 50–75% in extension. A schedule was planned for piperacillin–tazobactam (8 days ago) and methylprednisolone (1 mg/kg/day for 5 days, with progressive reduction) and prophylactic anticoagulation. Patient 6, who exhibited hypoxemia in both episodes, used hydroxychloroquine 400 mg, azithromycin 500 mg for 5 days, and levofloxacin 750 mg from the 6th to the 10th day. On the second visit, the patient was prescribed ivermectin 6 mg (two tablets, 24/24 hours) for 3 days, azithromycin 500 mg/day for 5 days, and prednisone 80 mg/day from the 6th to the 10th day.

## DISCUSSION

We report cases with recurrence of symptoms compatible with COVID-19, with positive RT-PCR results, that could represent the occurrence of viral reactivation or reinfection. The interpretation of this phenomenon is subject to questioning because the detection of the viral genome by RT-PCR has persisted in some cases for up to 6 weeks.^6^ The long asymptomatic period and characteristics of the symptoms reinforce the possibility of recurrence. However, our data are not sufficient to prove the occurrence of such phenomena, nor to characterize their nature.

Despite efforts to produce a vaccine, a licensed one remains unavailable.^12^ It is likely that health professionals with COVID-19 will require post-discharge and post-recovery surveillance of signs and symptoms, with retesting for SARS-CoV-2 for those who present recurrent clinical manifestations of the disease. We emphasize the importance of maintaining the use of personal protective equipment and hand hygiene even after curing COVID-19 when returning for assistance. Determining the possibility of reinfection or reactivation of the virus and immunological protection after natural infection has important implications for coping with the COVID-19 pandemic in the future.

One limitation found in the present investigation was the absence of cultures and sequencing of the virus, which were not possible. After the occurrence of these cases, the local government implemented surveillance by electronic means, with the objective of detecting earlier new suspected cases with this profile. In addition, longitudinal cohort studies will assist in better understanding the prognosis of the disease.
